# Malignant Catarrhal Fever of Cattle Is Associated with Low Abundance of IL-2 Transcript and a Predominantly Latent Profile of Ovine Herpesvirus 2 Gene Expression

**DOI:** 10.1371/journal.pone.0006265

**Published:** 2009-07-15

**Authors:** Claudia S. Meier-Trummer, Hubert Rehrauer, Marco Franchini, Andrea Patrignani, Ulrich Wagner, Mathias Ackermann

**Affiliations:** 1 Institute of Virology, Zurich, Switzerland; 2 Functional Genomics Center, University of Zurich, Zurich, Switzerland; Institute of Molecular and Cell Biology, Singapore

## Abstract

**Background:**

Malignant catarrhal fever (MCF) is a lethal disease of cattle, characterized by vasculitis, necrosis, and accumulation of activated, dysregulated cytotoxic lymphocytes in various tissues. Ovine gamma herpesvirus 2 (OvHV-2) is a causative agent of MCF, which may trigger the disease through immunopathogenic pathways. Lymphocytes are the main target of the virus. However, the pathogenic basis of the disease is still mysterious.

**Methods/Findings:**

We hypothesized that the gene expression patterns of OvHV-2 and the relative abundances of host cell transcripts in lymphnodes may be used to identify pathways that help to explain the pathogenesis of MCF. Therefore, viral and host cell gene expression patterns in lymph nodes of animals with MCF and healthy controls were analyzed by microarray. Two regions on the viral genome were transcriptionally active, one encoding an orthologue to the latency-associated nuclear antigen (ORF73) of other gamma herpesviruses, the other with no predicted open reading frame. A vast number of transcripts related to inflammatory processes, lymphocyte activation, cell proliferation and apoptosis were detected at different abundances. However, the IL-2 transcript was eminent among the transcripts, which were, compared to healthy controls, less abundant in animals with MCF. The ratio between CD4- and CD8-positive T-lymphocytes was decreased in the lymphnodes of animals with MCF compared to healthy controls. In contrast, the same ratio was stable, when peripheral blood lymphocytes were analyzed.

**Conclusions/Significance:**

The phenotype of mice with a deficient IL-2-system almost perfectly matches the clinical signs observed in cattle with MCF, which feature a significantly decreased IL-2 transcript abundance, compared to healthy cattle. This supports the hypothesis that immunopathogenic events are linked to the pathogenesis of MCF. IL-2-deficiency may play an important role in the process. Therefore, this work opens new avenues for research on MCF.

## Introduction

Malignant catarrhal fever (MCF) is a mysterious and lethal immunopathological disease of cattle and other cloven hoofed animals. Etiologically, MCF can be instigated by at least two distinct members of the Macavirus genus within the subfamily *gammaherpesvirinae*, i.e. alcelaphine herpesvirus 1 (AlHV-1) and ovine herpesvirus 2 (OvHV-2)[1]. Both viruses undergo subclinical infections in their natural reservoir hosts, whereas so-called indicator hosts, i.e. cattle, deer, bison, swine, succumb to MCF [2], [3], [4], [5], [6], [7], [8], [9], [10], [11]. OvHV-2 is asymptomatically endemic worldwide in all breeds of sheep, giving cause to the occurrence of sheep-associated MCF (SA-MCF), wherever sheep and indicator hosts are kept in close vicinity [12], [13], [14]. In contrast, AlHV-1 is asymptomatically endemic in African ungulates, for example wildebeest, and MCF due to AlHV-1 is restricted to African countries or may occur on other continents upon contact of susceptible animals with infected Zoo animals [15].

The disease is characterized by the infiltration and accumulation of large numbers of CD8-lymphocytes, causing vasculitis and necrosis in a variety of tissues [10], [16], [17], [18]. Various clinical patterns can be discriminated, i.e. a head-and-eye form, an intestinal form, and a cutaneous form [10], [19], [20]. The clinical findings may include combinations of ocular and nasal discharge, opacity of the cornea that may lead to blindness, diarrhea, haematuria, erosions of the muzzle, lymphnode swelling and eventually erosions on the skin [10], [16], [17]. However, the symptoms are often not clearly attributable to one of the clinical patterns, e.g. diarrhea occurs in nearly all affected animals.

While AlHV-1 has been isolated in the 1960ies and can be serially propagated in cell cultures, there is no suitable monolayer cell culture system to serially propagate OvHV-2 [2], [3]. Therefore, it has been difficult to study OvHV-2 and its underlying pathogenesis in either sheep or cattle. However, much progress has been achieved in recent years due to advances in molecular biological techniques. Initially, it was the detection and analysis of herpesvirus-like DNA in tissues from animals with MCF, which allowed consecutive establishment and improvement of various PCR detection and quantification methods for OvHV-2 [6], [21], [22], [23]. These technical developments allowed for studies on virological, epizootological, and pathogenetical aspects of OvHV-2 in various animal species and also for the establishment of a very useful rabbit model of the disease [24], [25], [26].

OvHV-2 exhibits typical features of a gamma herpesvirus, which has been confirmed through the recent completion of its genomic DNA analysis [27]. OvHV-2 has a double stranded DNA genome, which can be divided into a unique long fragment of approximately 130 kbp and multiple copies of approximately 4 kbp terminal repeat elements. The genome encodes for at least 73 open reading frames (ORFs), 62 of which show homology to known gamma herpesvirus genes. Among others, a gene encoding for a latency-associated nuclear antigen (LANA), a key feature of gamma herpesviruses, was predicted in ORF73. The other genes are either shared with AlHV-1 or unique to OvHV-2 (Table 1). Among this second set of genes, there are several candidates that could provide explanations for the disease phenotype, which includes uncontrolled multiplication of lymphocytes in various tissues. For example, a spliced homolog of cellular interleukin 10 (vIL-10, Ov2.5) has been described, which may serve as a growth factor for the host's lymphocytes and which also may act differently, depending on the animal species infected. Furthermore, two homologs to Bcl-2 (Ov4.5 and Ov9) have been identified. These might contribute to the protection of infected cells against intrinsic or extrinsic apoptosis, which is induced in the course of a normal immune reaction to prevent uncontrolled multiplication of activated lymphocytes (reviewed by [28]).

**Table 1:** Unique OvHV-2 genes.

Unique gene	Possible function
Ov2	Regulation of transcription; leucine zipper protein
Ov2.5	Viral IL-10 (vIL-10)
Ov3	Intracellular signaling; semaphorin family
Ov3.5	No prediction, unknown
Ov4.5	Cell death regulator; Bcl-2 family
Ov5	Intracellular signaling; G-protein coupled receptor
Ov6	Viral transactivator; similarity to Zta of EBV
Ov7	Glycoprotein
Ov8	Glycoprotein
Ov9	Cell death regulator; Bcl-2 family
Ov10	Transcriptional regulation; nuclear localization signal

The major sites harboring high amounts of viral DNA in the course of MCF comprise blood lymphocytes and organs of the immune system, including spleen and lymphnodes. However, the presence of viral DNA in each single dysregulated cell, which contributes to the disease picture, has been a matter of debate. Some authors believe that only a fraction of lymphocytes is infected, whereas others argue that the frequency of virus positive cells *in vivo* is being underestimated due to the lack of sensitive methods for detection [6], [24], [25], [29], [30].

Based on these arguments, we set out to test the following two hypotheses:

- Development of MCF is associated with increased survival and multiplication of latently infected lymphocytes, which are protected from apoptosis through functions of a specific set of viral proteins, including Ov2.5, Ov4.5, and Ov9 (Table 1). The expression of the corresponding viral genes in diseased animals can be measured by a viral microarray. Survival levels of infected cells could be increased through direct interaction of viral proteins with cellular proteins, which regulate apoptosis in activated lymphocytes. In this case, the gene expression patterns of the infected cells would not necessarily be different from those of uninfected cells. In the same assay, a predominantly lytic type of viral gene expression was expected to be recognizable.
- Alternatively, viral proteins or micro RNAs could influence the cellular gene expression patterns, which can be recognized through a microarray analysis of cellular gene expression. In this case, the pathogenesis of MCF could also be based on a dysfunctional interplay between the cells involved in immune functions. In such a model, only a fraction of relevant cells needs to be infected to allow for this type of pathogenesis. Furthermore, the pattern of viral gene expression may be distinct from that proposed in the first hypothesis.

While a lytic type of virus infection would be difficult to explain, in both alternative cases, the normal pathways to restrict multiplication of activated lymphocytes by induction of apoptosis would be disturbed, which could result in dysregulated multiplication of lymphocytes as a basis for the disease phenotype.

To test these hypotheses, we generated a microarray for the semi-quantitative detection of viral transcripts. Labeled cRNAs were tested on the viral microarray as well as on a cattle microarray comprising the relevant genes for analyzing the general features of the host's status of immune response. Important findings were corroborated by alternative methods. We found that, indeed, MCF was associated predominantly with a latent type of viral gene expression and, furthermore, we may have detected an important clue to understand and, possibly, treat MCF in the future.

## Results

Lymphnodes are one of the main sites for diagnosis of MCF and lymphocytes are the main carriers of OvHV-2 DNA in cattle with MCF. In order to get insight into the pathogenesis of this disease, an effort was undertaken to analyze the cellular and viral transcription profiles in such lymphnodes and to compare the cellular transcription profiles of animals with MCF to those of uninfected animals. For this purpose RNA was extracted from OvHV-2-positive lymphnodes of cattle with MCF as well as from OvHV-2-negative, healthy control animals. Consecutively, Cyanin- and biotin-labeled cRNA was produced for use in microarray analysis and standardized as described in Materials and Methods. Presence and quantity of selected viral RNAs was additionally assayed by qRT-PCR.

### Analysis of viral transcripts

#### Microarray

**Figure 1:**
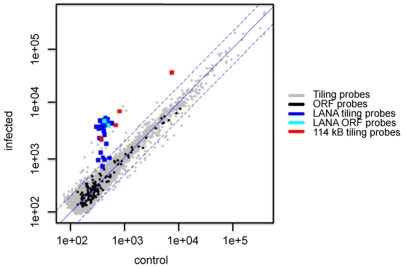
Hybridization signals. Comparison of the hybridization signals of infected (MCF-diseased) and control animals on the viral microarray. The tiling and ORF specific probes are plotted in gray and black. We have highlighted the probes matching the LANA homologue (blue/cyan) and 4 consecutive tiling probes targeting the region 115250 bp on the forward strand (red).

**Figure 2:**
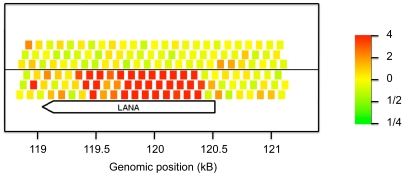
OvHV-2 gene expression. Expression pattern in the region 119 kB to 121 kB of the virus. The plot shows the expression changes measured by the probes tiled across the genome and the predicted location of the LANA gene. Probes above (below) the dividing line match the forward (reverse) strand. Significant consistent induction is measured by the probes matching the reverse strand at the locus of ORF73 (LANA orthologue).

In a first set of experiments, the Cy3- and Cy5-labeled cRNA was used for hybridization with an array, which represented the entire OvHV-2 genome. Specifically, 8730 60mer oligonucleotides were used as viral targets. These oligonucleotides represented both strands of the viral genome twice (tiling probes). To generate a second type of screening for transcriptional activity, two additional oligonucleotides per each predicted viral open reading frame (ORF) were used as targets, which had been selected for optimal hybridization under the conditions used (ORF probes). The array was hybridized and analyzed as described in Materials and Methods. The resulting data were deposited in the GEO database (GSE13853). Figure 1 compares the hybridization signals obtained from cells of infected and control animals. The points represent the expression signal of the probes targeting the viral genome. The majority of the probes (gray and black points) did not show any differential signal. Only two regions on the virus genome showed transcriptional activity. The first one covered ORF73, which is located on the reverse strand spanning the region 119046 bp to 120533 bp and represents a LANA orthologue. In this region 31 consecutive tiling probes showed differential signals in the infected animals (see Figure 2). The same was also observed by the two probes that were optimized to target the ORF73 (cyan dots in Figure 1). Thus, transcription from ORF73 was detected using two alternative methods.

The second region for viral transcriptional activity spanned nucleotides 115184 to 115364 bp on the forward strand of the viral genome and was represented by four consecutive tiling probes. This region corresponds to an intergenic region, located between ORF69 and ORF Ov8. This observation was unexpected and invites for further analysis.

#### qRT-PCR

The present microarray was designed only to provide relative information towards viral transcriptional activity throughout MCF. In order to validate the presence of the transcripts predicted by the viral microarray and to explore the sensitivity of the present assays, a number of quantitative reverse transcription real-time PCR (qRT-PCR) assays were established (see Materials and Methods) and used for detection and quantitation of selected viral RNAs in lymphnodes of cattle with MCF. The results are summarized in Table 2. Briefly, the sensitivity of the assay to detect ORF25 template was comparable to that for detecting the intergenic target, whereas the tests to detect ORF73 and Ov9 were more sensitive. Under these conditions, the ORF25 transcript, encoding the major capsid protein of OvHV-2, remained undetectable in our materials, whereas the intergenic transcript was present at high numbers. Similarly, between 100 and 1.000 copies of the ORF73 transcript were detected using the same amount of template that did not reveal any transcriptional activity over Ov9. Calculating conservatively, the microarray used throughout our experiments had a detection limit of less than 100 copies per assay.

**Table 2:** qRT-PCR for selected viral RNAs in lymphnodes.

Target	DNA^a^	Melting peak^b^	RNA (MCF)^c^	RNA (healthy)^d^
ORF25	100–1000	n.a.	Not detected	Not detected
ORF73	>10	81°C	100–1.000	Not detected
Ov9	>10	81°C	Not detected	Not detected
Intergenic	>100	83°C	500–10.000	Not detected

a detection limit (number of template copies) using DNA template.

b determined following qPCR using SYBR-green technology; not applicable (n.a.) to conventional PCR, which was detected by EtBR staining after agarose gel electrophoresis of the PCR product.

c 25 ng RNA template (from lymphnodes of animals with MCF) before reverse transcription. Numbers in the column refer to copy number detected.

d 25 ng RNA template (from lymphnodes of healthy animals) before reverse transcription.

From all of the above experiments we conclude that no transcripts corresponding to structural viral proteins were detected. These results supported the notion that MCF was associated with a predominantly latent OvHV-2 infection. However, they also clearly, contradict hypothesis 1, which predicted that several viral genes would be expressed, which are unique to OvHV-2 or shared with AlHV-1 (see Table 1). Indeed, no significant transcriptional activity in any of those genes was detected under the present experimental conditions.

### Analysis of host transcripts in animals with and without MCF

The biotin-labeled cRNAs were also used for hybridization with an Affymetrix Bovine Genome array, which consisted of 24.072 probe sets, each comprising 11 oligonucleotides, covering over 23.000 bovine transcripts. 15.425 probe sets were considered as present by our filtering. The subsequent statistical analysis revealed significant expression differences (p<0.05) between infected and uninfected animals for 6.300 probe sets. The resulting data were submitted to the GEO database (GSE13852). Apparently 4.538 transcripts were significantly more abundant in diseased animals as compared to healthy animals. Furthermore, 1.770 transcripts were significantly less abundant in animals with MCF. 1.238 transcripts were found to be more than twice as abundant in diseased than in healthy animals. The highest value of higher abundance (256-fold) was found with a transcript for granzyme-2, a T-cell serine protease, which is transcriptionally activated during cytotoxic T-lymphocyte maturation. The detailed data were deposited in the GEO database (GSE13852).

#### Inflammation and T-cell activation

As expected from the etiology and the clinical disease picture, primarily transcripts related to inflammatory processes, lymphocyte activation, catalytic processes, immune response, cell proliferation and apoptosis were detected at different abundance. Unexpectedly, the IL-2 transcript was eminent among the transcripts of low abundance. Since IL-2 is strictly regulated on the transcriptional level [31] and because it occupies a pivotal role in the regulation of the immune response and due to the fact that IL-2 deficient mice show a similar phenotype to cattle with MCF, i.e. accumulation of lymphocytes in the intestine and ulcerative colitis [32], [33], we suggest that its low abundance in the context of inflammation and T-cell activation may represent a key feature of MCF.

The transcripts for the IL-17 receptor, MHC-I heavy chain, IL-6 receptor alpha chain, insulin receptor, and IL-16 receptor were among the most strongly reduced transcripts. On the other hand, the expression of IL-10 and its receptor were slightly increased. An interesting phenomenon was observed with various clusters of the T-cell receptor (TCR). While the TCR beta cluster (Bt63956) was in low abundance, other beta clusters as well as the gamma cluster were significantly increased (Table 3). A number of transcripts belonging to the effector molecules of cytotoxic T-cells, e.g. granzymes and perforin, as well as transcripts indicating the lymphocytes to be activated, e.g. CD2, CD3, CD8 were detected at higher abundance. The Interferon gamma transcript itself was 6.4 times more abundant in animals with MCF, and in accordance with this we observed that among 21 interferon-related transcripts, 20 were found to be more abundant and only 1 was less abundant. The outstanding higher abundance of transcripts belonging to catalytic processes may be explained by the predominance of cytotoxic T-cells in MCF lesions.

**Table 3:** Transcripts associated with inflammation and T-cell activation affected in lymphnodes of MCF affected cattle.

Transcript	Fold abundance	p-value	Function
Granzyme A	8.6	0.000372	Cytotoxic T-cell effector
Granzyme B precursor	256.2	0.0175	Cytotoxic T-cell effector
Granzyme H precursor	27.2	0.000431	Cytotoxic T-cell effector
Perforin	4.8	0.00517	Cytotoxic T-cell effector
CD2	2.4	0.00569	Adhesion molecule involved in T-cell activation
CD3ε	1.7	0.00517	Essential role in TCR signal transduction and cell-surface expression of the TCR
CD3δ	1.7	0.00837	
CD3γ	1.5	0.00838	
CD8α	3.9	0.00598	Coreceptor for MHC class I restricted T-cell s
CD28	2.2	0.00161	Costimulation of T-cell proliferation and cytokine production upon binding CD80 or CD86
TCR, gamma cluster	4.8	0.0457	Recognition of antigen presentation
TCR, beta cluster (Bt63956)	0.4	0.0364	Recognition of antigen presentation
TCR, beta cluster (Bt1978)	1.4	0.0126	Recognition of antigen presentation
TCR, beta cluster (Bt1978)	1.4	0.00852	Recognition of antigen presentation
Interferon gamma	6.4	0.00958	Affects activation, growth, and differentiation of T-cells, B-cells and macrophages as well as Nk cells. Upregulates MHC expression on APCs. Antiviral and anti-proliferative activities
Interleukin 2	0.14	0.00268	Involved in propagation and establishment of self tolerance of T-cells
Interleukin 7	0.6	0.0277	Growth factor for T-cell progenitors
Interleukin 10	2.9	0.0132	Anti-inflammatory cytokine
IL-10 receptor	1.3	0.007	Anti-inflammatory response

#### Cell cycle/Apoptosis associated transcripts

Several cell cycle and apoptosis related transcripts were present at different abundance in healthy and MCF-diseased cattle, respectively (Table 4). Interestingly, the transforming growth factor beta (TGF beta) as well as its receptor (TGFbR), which together control proliferation and differentiation of many cell types, was less abundant in animals with MCF. This is noteworthy since TGF beta is, similar to IL-2, important in the context of regulatory T-cells. The complexity of the present situation may be explained by the simultaneous influence of the virus and host control mechanisms on affected lymphocytes.

**Table 4:** Cell cycle and apoptosis related transcripts affected in MCF in cattle.

Transcript	Protein family	Fold induction	p-value
Cyclin A2	Cyclins	4.2	0.000587
Cyclin B1		7.3	0.000159
Cyclin B2		5.3	0.00247
Cyclin E2		3.7	0.0015
Fas ligand	Membrane bound cytokine	5.1	0.0031
Fas	Tumor necrosis factor	1.4	0.0045
Cyclin dependent kinase 2		2.2	0.00114
Cyclin dependent kinase 5		1.7	0.00458
Cyclin dependent kinase 7		1.5	0.0158
Cyclin dependine kinase 11		1.4	0.0375
BH3 domain interacting death agonist (BID)	B-cell lymphoma type 2 (Bcl2)	2.2	0.000862
Bcl2-associated X protein (BAX)		2.9	0.00503
Bcl2-like 7 (BAK)		2.3	0.00275
Bcl2		1.6	0.00159
Voltage-dependent anion channel (VDAC) (3 probe sets)		2.2	Probe set 1 = 0.00077
		2.0	Probe set 2 = 0.00142
		1.6	Probe set 3 = 0.00186
Baculoviral IAP repeat containing 5 (survivin)	Inhibitors of apoptosis (IAP)	6.3	0.00326
Siva		2.1	0.000791
T-cell specific tyrosine-protein kinase (LCK)	Tyrosine-protein kinase	1.9	Probe set 1 = 0.000703
		2.0	Probe set 2 = 4.23×10^−5^
		1.8	Probe set 3 = 3.28×10^−5^
Apoptosis-associated speck-like protein containing a CARD (caspase recruitment domain)		2.4	0.00248
Hsp10	Heat shock proteins (Hsp)	2	0.00158
Hsp70		0.34	0.00833
Hsp90		1.8	8.2×10^−5^
Retinoblastoma protein (Rb) (2 probe sets)		2.2	9.96×10^−5^
		1.5	0.004
E2F	Transcription factor	2.2	5.7×10
p53	Transcription factor	1.3	0.00134
TGF beta	TGF beta family	0.29	0.006
TGF beta receptor		0.35	0.01

Overall, these results were consistent with hypothesis 2, which claimed that the host gene expression of animals with MCF was affected in a manner that could be detected by microarray analysis and that could explain the disease phenotype without all dysregulated cells being infected. However, it remains to be clarified, whether these observations are caused by differential gene expression regulation of the cells or by their mere numbers and abundances.

### CD4/CD8 ratios

**Figure 3:**
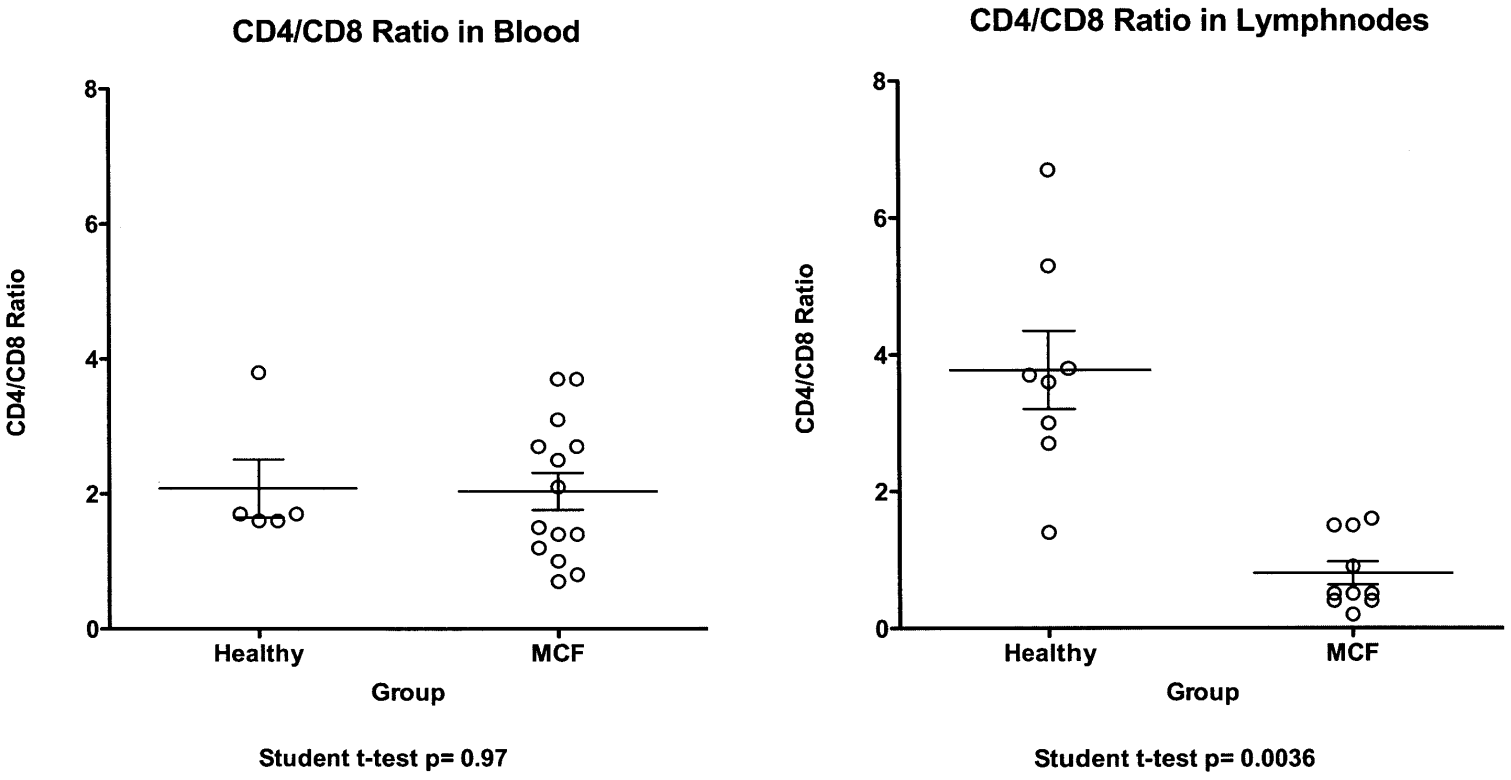
CD4 to CD8 ratios. The CD4 to CD8 ratios (y-axis) in blood (left panel) and inguinal lymphnodes (right panel) of healthy animals and cattle with MCF are shown. Open circles indicate individual values, horizontal bars give median values, and standard deviations are shown by vertical brackets.

The relative abundance of transcripts in a given compartment may be explained either by downregulation/upregulation of transcriptional activity or by loss/proliferation of the major producer cells of a particular transcript. To address this issue as far as possible, CD4/CD8 ratios were determined in the peripheral blood as well as in the lymphnodes of cattle with MCF and healthy controls. As shown in Fig. 3., the CD4/CD8 ratios in the bloodstream did not significantly differ between healthy animals and such with MCF (p = 0.97). In contrast, the same ratios were significantly lower in cattle with MCF compared to healthy controls (p = 0036), when assayed in lymphnodes. Due to the nature of the lymphnode, absolute counts of the relevant cells could not be generated. Therefore, the observed low abundance of IL-2 transcripts in lymphnodes of cattle with MCF might be interpreted either as due to (1) over-proportional proliferation of CD8+ cells and downregulation of IL-2 transcription in CD4 cells or (2) strong decline of the CD4+ cells, which are the main IL-2 producers, or (3) a combination thereof. However, it was obvious that the number and fate of lymphocytes in the lymphnodes may differ from the situation in the periphery.

In any event, our results strongly suggest that the low abundance of IL-2 transcripts in lymphnodes of cattle with MCF may lead to IL-2 deficiency and be an important factor in the pathogenesis of the disease.

## Discussion

Malignant catarrhal fever (MCF) in cattle is a frequently lethal disease, which proceeds with fever, depression, conjunctivitis and keratitis, as well as with hyperemic to ulcerative lesions in the mucosa of the respiratory, genital and digestive tract, which lead to ocular and nasal discharge and diarrhea [11]. Viral DNA can be detected in buffy coat cells of cattle with MCF as well as in most organs. In contrast, demonstration of viral antigen in cattle with MCF has, thus far, been largely unsuccessful [2], [3].

At least two agents, AlHV-1 and OvHV-2, are known to cause MCF in cattle and it would be of interest to know, whether or not their disease is actually based on the same pathogenetical principles. In this work, we concentrated exclusively on OvHV-2. Similar to other gamma herpesviruses, OvHV-2 has two sets of genes, i.e. (1) a set of common viral genes that are conserved among the herpesviruses and (2) a set of unique genes that often have homologues in their host cells. Twelve (Table 1) of its 73 predicted ORFs encode for unique genes, nine (Ov2, Ov3, Ov4.5, Ov5, Ov6, Ov7, Ov8, Ov9, Ov10) have orthologues in AlHV-1 and three are exclusive for OvHV-2 (Ov2.5, Ov3.5, Ov8.5).

It is well known from other gamma herpesviruses that the viral gene expression pattern may correlate to the present state of the infection or the associated disease. For example, the latency II program of EBV drives the differentiation of activated B-cells into memory cells [34], [35] or vGCR of HHV-8 is known to induce transformation and angiogenesis, both features, which are required for the development of Kaposi's sarcoma [36], [37], [38], [39].

Although the pattern of viral gene expression in infected cells of animals with MCF has previously not been analyzed in detail, a recent report described the detection of the ORF25 transcript, which encodes for the OvHV-2 major capsid gene, in animals with MCF [40]. This observation spoke for lytic replication of OvHV-2 in diseased animals, at least upon infection with an American OvHV-2 strain. In contrast, we were unable to detect the ORF25 transcript throughout our work, which, though, was done in the context of European strains of OvHV-2. Interestingly, the ORF25 transcript was also not detected, when analyzed by others in the rabbit model for AlHV-1-associated MCF [41]. The latter authors explicitly did not detect any ORF25 (capsid protein) or ORF9 (DNA polymermase) transcripts in spleens or lymphnodes of rabbits with AlHV-1-induced MCF. They concluded, therefore, that AlHV-1-induced MCF is associated with a predominantly latent infection. This view is also shared by others working with European OvHV-2 [42]. Indeed, mainly intact circular OvHV-2 genomes have been found in T-lymphocytes derived from cattle with MCF, which speaks for a dominating latent infection. Furthermore, detection of structural viral antigens in the lesions of cattle with MCF was, hitherto, unsuccessful. Moreover, OvHV-2 is rarely, if at all, naturally transmitted from one cattle to another and it has been difficult to recover infectivity at all from cattle with MCF [43], [44], [45]. Conversely, a mixture of OvHV-2 transcripts has been detected in cultured peripheral T-lymphocytes from cattle with MCF [29]. Those authors concluded that, at least in the periphery, latently infected cells may co-exist with cells harboring productively replicating OvHV-2. This view is supported by a recent publication on OvHV-2-induced MCF in the rabbit model, where structural OvHV-2 proteins were detected exclusively in epithelial cells and M-cells of the appendix, while other tissues contained viral DNA at detectable levels but not the corresponding mRNAs or proteins [26].

However, in lymphnodes of cattle with MCF, as demonstrated throughout our experiments, neither genes encoding for structural proteins nor any one of the unique genes of OvHV-2 were detected above threshold levels. Indeed, the only active gene seemed to be ORF73. Detection of this RNA was confirmed in our work by three different approaches. Interestingly, ORF73 (LANA) transcripts were also detected in rabbits with AlHV-1-induced MCF [41]. Thus, our data with OvHV-2 in cattle match very well with the observations described for AlHV-1 in rabbits. Together, these data imply that MCF, independent of its individual agent or host, is associated with a predominantly latent viral gene expression pattern, at least in certain tissues.

In addition, we detected by microarray a transcriptional activity in a region without any predicted ORF. The existence of this transcript was confirmed by qRT-PCR over the same region (Table 2). Although the sensitivity of the qRT-PCR for this transcript was below that for ORF73, the number of molecules covering the intergenic region was higher than the number of ORF73 transcripts. Since the detection limits of the assays for ORF25 and intergenic RNA were comparable, the detection of the latter RNA also corroborates the absence of the former.

While a latency pattern, rather than a replicative pattern, of viral gene expression could be expected in the context of MCF, the lack of expression from the atypical genes argued against our first hypothesis, which claimed that some of those genes and their products might directly drive survival of the dysregulated cells. One might predict that those genes and their products have a function in protecting their original host from developing MCF. Absence of their expression in cattle could even be one of the major reasons for the development of MCF. Unfortunately, OvHV-2 gene expression could not yet be determined in ovine cells, primarily due to the scarcity of infected cells in sheep (own unpublished observations).

### Host gene expression

The host gene expression profile was markedly disturbed by the effects of the disease. One may argue, whether or not the lymphnodes are the ideal tissue to look for viral and host gene expression throughout MCF. They were selected for the present study because they are known to harbor plenty of OvHV-2 infected cells, which play an important role in the host's immune defense [10], [22]. Moreover, the vasculitis and necrosis in affected tissue have been attributed to the function of riotous cytotoxic lymphocytes. It was not surprising to find a complex pattern of differences in transcript abundances upon comparison of transcriptomes in lymphnodes from healthy cattle and cattle with MCF.

### High abundance of Cyclin and Cdk transcripts

The state of cell proliferation can be monitored through analysis of gene expression and was reflected on the present microarray. As the cells progress through the reproductive cycle, cyclins are synthesized and degraded. Cyclins bind to and, thus, activate cyclin-dependent kinases (Cdks). Activated Cdks induce expression of more cyclins as well as degradation of the Cyclin-Cdk inhibitor of the consequent step in the cell cycle. In contrast to cyclins, Cdk levels remain, under normal conditions, constant throughout the cell cycle. However, cyclin-dependent kinases are frequently upregulated in malignancies due to overexpression of their cyclin partners [46]. Thus, as a first marker of malignancy, a significant higher abundance of several cyclins as well as Cdk was detected in the lymphnodes of cattle with MCF (Table 4). This finding could be expected since histology of lymphnodes in MCF diseased cattle is dominated by a marked increase in lymphocytes [47], although foci of necrosis are concurrently detected [16], [17].

### Activation of T-cells and unbalanced expression of regulators of apoptosis

T lymphocyte activation can be expected upon a viral infection of an organism or during defense against uncontrolled cell growth. Indeed, several markers of T lymphocyte activation were more abundant in cattle with MCF as compared to healthy animals (Table 3). To avoid accumulation of excessive amounts of activated T-cells, both the extrinsic and the intrinsic apoptosis pathways will usually be induced, simultaneously with T-cell activation. Both pathways lead to the activation of a family of cystein proteases called caspases. Caspases are constitutively present in most cells as inactive proenzymes and are activated by specific proteolytic cleavage. On a transcriptomic microarray, changes in apoptosis levels will therefore likely be reflected by changes in the levels of transcripts of apoptosis regulatory proteins without directly affecting the transcript levels of the caspases themselves. In the present study, the expression of antiapoptotic members of the Bcl-2 family as well as other inhibitors of apoptosis (IAP), were more abundant in cattle with MCF than in healthy animals (Table 4).

However, opposing forces, stimulating cell death, were also observed in lymphnodes of cattle with MCF. Death receptors are cell surface receptors that send apoptosis signals to the inside of the cell when they are bound by death ligands. Death receptors are members of the tumor necrosis factor (TNF) receptor gene superfamily. They all contain a homologous cytoplasmic sequence named death domain. The best characterized death receptors and corresponding death ligands are Fas (Fas and its ligand FasL, also called CD95 and CD95L) and TNF (TNFR1 and its ligand TNF) [48]. T-cell receptor (TCR) engagement, i.e. activation of T lymphocytes leads to the expression of Fas (CD95) on the surface of the activated T-cell, a prerequisite to undergo activation induced cell death (AICD) [49], [50]. Binding of FasL to Fas results in trimerization of Fas, which includes the approximation of death domains in the cytoplasmic tails of Fas. In the present study, the FasL as well as Fas transcripts were detected at significantly higher abundance in cattle with MCF (Table 4). FasL activity was previously described to be induced predominantly at the transcriptional level [51], [52], [53]. CD27 is another member of the TNF receptor family expressed on B and T-cells. Siva, which was found to be highly abundant in the context of MCF (Table 4), is an intracellular ligand of CD27. It is expressed in lymphoid cells and exhibits proapoptotic activity [54], [55].

In addition, some tumor suppressor genes, i.e. retinoblastoma protein (Rb) and LCK as well as the proapoptotic p53 were more abundant in lymphnodes of cattle with MCF than in healthy cattle (Table 4). Rb is known to arrest cells in the G1 phase of the cell cycle by binding to E2F transcription factors (Reviewed by [56], while free E2F factors are important for the induction of S phase entry. Complexes of Rb family members and E2Fs recruit histone deacetylase and other chromatin remodeling factors to E2F-responsive promoters and therefore inhibit transcription from the same [57], [58], [59]. However, E2F transcripts were also more abundant, which might counter balance the arresting effect of Rb.

In T lymphocytes protein tyrosine kinases (LCK) play an integral role in the activation of cells through various immunoreceptor molecules [60], [61]. Apart from the activation of the cell cycle, LCK have been shown to be involved in programmed cell death of T lymphocytes. Gonzalez-Garcia et al showed an induction of CD95 ligand through LCK [62]. Samraj et al showed a positive regulation of the mitochondrial apoptosis by LCK [63]. In our experiment LCK was highly more abundant in the context of MCF.

Thus, both pro- and anti-apoptotic forces seemed to be induced in cattle with MCF, which might lead to a pathogenic alteration in the natural balance between cell survival and cell death.

### Low abundance of the IL-2 transcript may explain the disease phenotype

IL-2 is used as an essential factor for the propagation of T-cells in culture [64]. Based on this property, IL-2 was also used for the augmentation of T-cell responses in vivo in cancer or AIDS patients [65], [66]. Importantly, IL-2 is strictly regulated at the mRNA level, which depends on signaling from the TCR and CD28. The suitability of both, microarray and real-time RT-PCR, for determining the levels of IL-2 mRNA in bovine cells and the good correlation of the two methods has been confirmed in a different context by others [67]. In our analysis, a significant low abundance of the IL-2 transcript was noticed in cattle with MCF, while CD28 and TCR transcripts were present at higher than normal abundance. Since stimulation through CD28 in addition to the TCR can provide a potent co-stimulatory signal to T cells for the production of various interleukins (IL-2 and IL-6 in particular), these observations imply that MCF is associated with a low abundance of the IL-2 transcript.

However, the low abundance of the IL-2 transcript might be due either to down regulation of its expression or to the loss of IL-2 producer cells. It was, therefore, interesting to note that the ratio of CD4+ to CD8+ T-cells in lymphnodes of cattle with MCF was decreased when compared to healthy controls. Interestingly, this same change has been observed in the AlHV-1-based rabbit model [41]. Yet, there, the change was associated to an increased growth of CD8+ T-cells, which in turn cannot explain any low abundance of the IL-2 transcript. Therefore, we like to speculate that down regulation of IL-2 transcription may at least be partially responsible for low abundance of the IL-2 transcript in cattle with MCF.

Of note, the ratios between CD4+ and CD8+ T-cells were unaffected in the periphery, when analyzed in the bloodstream of cattle. This was in contrast to the observations made in rabbits with AlHV-1-induced MCF [41]. This is inasmuch important that the ratio measured in the periphery does not necessarily reflect the picture that is found in the lymphoid organs.

Interestingly, mice lacking a functional IL-2 system develop largely normal until the age of 4 to 6 weeks, where they start to suffer from polyclonal expansion of T- and B-cells. This expansion causes enlargement and non-purulent inflammation of lymph nodes, spleen, and gut-associated lymphoid tissue due to accumulation of activated T-cells. Similarly, a human patient with IL-2 receptor deficiency showed also signs of T-cell abnormalities as evidenced by lymphadenopathy, chronic inflammatory disorders, and lymphocytic infiltration of multiple organs [68]. Furthermore, T-cells from mice lacking either IL-2 or the IL-2 receptor have been reported to be resistant to activation-induced cell death in vitro and in vivo. Alternatively, it has been proposed that the abnormal growth rate of T-cells in association with IL-2 deficiency may be due to tolerogenic properties of IL-2, mediated through interactions with regulatory T-cells (Treg)[69], [70], [71]. Since IL-2 in the lymphnode is critical for the development and peripheral expansion of Treg (CD4+CD25+), which promote self-tolerance by suppressing autoreactivity of T-cells as well as limiting T-cell replication in vivo (reviewed by [31], decreased IL-2 levels may explain the accumulation and autoreactivity of the T-cells in MCF. In any event, the disease signs and abnormal T-cell properties seen in mice and men without functional IL-2 system are very reminiscent of the phenotypes associated to MCF in cattle. Thus, it seems that lack of IL-2 may play an important, if not central role in the development of MCF.

These observations instigate hope that it might be possible to treat MCF in cattle by supplementing IL-2. One might argue that it would be desirable to measure the peripheral IL-2 concentrations in order to support or reject this hypothesis. However, it has to be kept in mind that IL-2 functions mainly in the lymphnodes and peripheral IL-2 concentrations do not necessarily correctly reflect the micro situation in the lymphnode. Indeed, it has been shown by others that IL-2 supplementation may have considerable effects on the immune responses, while measurement of peripheral IL-2 expression and applied amount of external IL-2 were not a good indicators for its function *in vivo* [72], [73].

### Potential roles of OvHV-2 proteins and transcripts in the development of MCF

Latency-associated nuclear antigens (LANA of HHV-8 or SaHV-2 and EBNA-1 of EBV) have been shown to play important roles in the development of gamma herpesvirus-associated neoplastic diseases in humans and other primates. However, their mode of action is associated with the expression levels or the functions of some major protooncogenes. For example, LANA of HHV-8 may either repress transcription of p53 [74] or direct the p53 protein to proteasomal degradation [75]. Furthermore, it can bind and inactivate retinoblastoma protein (Rb), thereby transactivating E2F transcription [76]. Similarly, SaHV-2 LANA can interfere with p53 or Rb functions, while EBV EBNA-1 interferes with p53 and HAUSP (Herpesvirus associated ubiquitin-specific protease) [77].

OvHV-2 ORF73 has been predicted to encode for a LANA orthologue, although its function has not yet been demonstrated [27]. LANAs are supposed to bind to the origin of latent viral DNA replication (oriP) and tether the viral DNA to the host's chromosome in order to allow co-replication of the viral genome with the cellular genome upon mitosis. For this purpose, any typical LANA orthologue needs to have DNA-binding properties, which might explain interference with the host's gene expression profile. Thus, binding of the LANA protein to any locus within the host's chromosome, including the IL-2 locus, might affect the patterns of gene expression, including IL-2 expression. Alternatively, the LANA protein might undergo interactions with host proteins, similar to the interactions described for its orthologues in other viruses. It will be interesting to analyze these possibilities in the future.

The second transcriptionally active region detected in cattle with MCF spanned nucleotides 115184 to 115364 bp on the forward strand of the viral genome. At present, one may only speculate about the nature and significance of this transcript. According to our results from qRT-PCR, this signal is attributable to one transcript that may even further extend into both directions. Although no STOP codon is evident in the +3 reading frame within the minimal boundaries of this transcript, it does not necessarily represent a hitherto undetected gene. According to its sequence, it may form a hairpin structure, which can be found in micro RNAs. Thus, it may be similar to BARTs and EBERs of EBV or to micro RNAs, which have been described for other viruses, including many herpesviruses [78], [79], [80], [81]. Such RNAs might interfere with the host's gene expression through mechanisms like siRNA or other means of silencing.

It will be interesting to address these issues in consecutive studies. Furthermore, it remains to be established, whether or not these observations are also true for MCF in animals other than cattle.

### Conclusions

We have determined the major viral gene expression pattern in lymphnodes of cattle with MCF. Only two sites were transcriptionally active, one with the potential to express ORF73, a LANA-orthologue, the other with some likelihood to represent a thus far unrecognized OvHV-2 gene or, maybe, miRNAs. Overall, this viral gene expression profile is similar to the one found in the context of AlHV-1-associated MCF in the rabbit model [41]. The prominent counterpart to this latent gene expression pattern on the host's side was a significantly lower abundance of IL-2 transcripts, an increase in lymphocyte activation, as well as increase and decrease in apoptosis associated transcripts in lymphnodes of cattle with MCF as compared to the same tissue from healthy animals. Taken as a whole, these results were consistent with one of our hypotheses, which claimed that the host gene expression of animals with MCF was affected in a manner that could be detected by microarray analysis and that could explain the disease without all dysregulated cells being infected. Since the phenotype of mice with deficient IL-2-system almost perfectly matches the colitis observed in cattle with MCF, we suggest that OvHV-2-linked low abundance of IL-2 transcripts may be a key to further study the pathogenesis of MCF. Clearly, in the present study, we did not discriminate between intracellular regulation of IL-2 mRNA expression and relative depletion of the lymphnode to harbor IL-2 producing cells. This important differentiation will have to be addressed in future studies. However, MCF may be looked at as an infectious form of IL-2-deficiency, which occurs as a natural disease of animals.

## Materials and Methods

### Animals

Mediastinal lymphnodes were taken from cattle at the slaughter house (11 healthy controls) and from 6 naturally diseased animals originating from conventional Swiss cattle farms, which had to be euthanized due to MCF. In addition, EDTA blood samples as well as inguinal lymphnodes were collected from 10 cows with and 8 cows without MCF.

### Construction of the OvHV-2 Agilent custom 11 k microarray

Based on the published sequence of OvHV-2 [27] we designed an oligonucleotide microarray for the analysis of viral gene expression in MCF. 60mer probes were chosen to match stretches along the entire genome [27] starting at genomic position 823 with an interval of 30 nucleotides, such that every position was covered by two probes. This was done for the forward as well as for the reverse strand. Further, we selected two more probes for each predicted open reading frame (ORF) of OvHV-2. These probes were designed using the software Arraydesigner (Premier Biosoft, Palo Alto, USA) and chosen such that the probe length was between 55 and 60 nucleotides and the predicted melting temperature was between 75°C and 80°C. The chip description was deposited at GEO database (GPL7746). Only probes with a quality score of ‘good’ were used. Altogether, we designed 8.876 probes targeting the viral genome. Additionally, we included 1500 oligonucleotides corresponding to known cattle genes for primary normalization of the hybridization intensities. Using our probe sequences, we ordered 11 k custom microarrays from Agilent. Microarrays were produced by Agilent by *in situ* synthesis technology.

### Affymetrix microarray for bovine transcription profile

We used the GeneCHIP® Bovine Genome array (Affymetrix., P/N 900561) for the analysis of the host gene expression profile.

### Total RNA preparation

Total RNA was isolated from lymph nodes of 9 cattle, two of which were sacrificed due to MCF, the others were MCF negative. Virus status of all animals was confirmed by real-time PCR. Lymph node tissue was frozen in liquid nitrogen and homogenized using a mortar and pistil. Total RNA was isolated using the RNeasy Kit by Qiagen (order number 74106, RNeasy, Qiagen, Hombrechtikon, Switzerland). Immediately upon homogenization the samples were taken up in RLT buffer with 1% β-mercaptoethanol (Sigma, Buchs, Switzerland). DNA was digested using RNase-free DNase (Qiagen, order number 79254) at room temperature for 15 minutes. RNA concentration was measured using a Nanodrop 1000 (NanoDrop Technologies, Delaware, USA). The quality of each sample was checked by a Bioanalyzer 2100 (Agilent, Waldbronn, Germany). Only those samples with 260 nm/280 nm ratio between 1.89–2.13 and a 28 S/18 S ration within 1.5–2 were further processed.

For the purpose of RT-PCR and qRT-PCR, the extracted RNAs were additionally subjected to removal of contaminating DNA using the Ambion Turbo DNA-free Kit (Applied Biosystems, Rotkreuz, Switzerland).

### Fluorescent cRNA preparation for the Agilent microarray (viral gene expression profile)

1.6–5 µg of total RNA were reverse transcribed to cDNA and amplified and labeled to cRNA with the Agilent Low RNA Input Linear Amplification Kit PLUS (order number 5184–3525, Agilent). Briefly, 1.2 µl T7 Promoter Primer and 1 to 5 µg of total RNA in a total volume of 11.5 µl were denatured at 65°C for 10 minutes. Then the reaction was placed on ice for five minutes. After that 8.5 µl cDNA master mix consisting of 4 µl 5× first strand buffer, 2 µl 0.1 M DTT, 1 µl 10 mM dNTP mix, 1 µl MMLV reverse transcriptase and 0.5 ul RNaseOUT were added to each sample and incubated at 40°C for 2 hours. Subsequently the enzyme was heat inactivated at 65°C for 15 minutes. Then the samples were placed on ice for five minutes. For the synthesis of fluorescent cRNA 2.4 µl Cyanine 3 or Cyanine 5-CTP (10 mM) and 57.6 µl transcription master mix consisting of 15.3 µl nuclease free water, 20 µl 4× transcription buffer, 6 µl 0.1 M DTT, 8 µl NTP Mix, 6.4 µl 50% PEG, 0.5 µl RNaseOUT, 0.6 µl inorganic pyrophosphatase and 0.8 µl of T7 RNA Polymerase were added and the reaction was incubated at 40°C in the dark for 2 hours. Labeled cRNA samples were isolated again with the RNeasy Kit (Qiagen).

### Hybridization of Agilent microarrays

From each positive animal, a Cy3- and a Cy5-labeled RNA sample, respectively, was co-hybridized with a reference sample with the opposite labeling and used as dye-swap pairs. As reference, the pool of the seven OvHV-2 negative samples was used. The specific activity of all samples as concentration of dye (pmol/µl) divided by concentration of RNA (µg/µl) was calculated. For fragmentation and hybridization we used the Agilent Gene Expression kit for oligo microarrays (Agilent, order number 5188–5242, protocol G4140-90050). Before hybridization the chips were blocked using the 10× blocking agent provided with the kit. 350 ng of Cy3 labeled RNA and 350 ng of Cy5 labeled RNA were mixed with 20 µl 10× blocking solution (provided with the kit), 4 µl 25× fragmentation buffer and toped up with nuclease-free water to 100 µl. Fragmentation was performed at 60°C in a hybridization oven (Agilent, G2545A) in the dark for 30 minutes. 100 µl 2× hybridization buffer was added to each tube and the microarray and gasket slide were assembled. The slides were hybridized at 65°C, for 17 hours at 4 rpm in a hybridization oven. After the hybridization, slides were washed twice for 1 minute in wash solution 1 (6× SSPE (Sigma, order number S2015), 0,5% N-lauroylsarcosine (Sigma, order number L7414) in de-ionised, nuclease-free water). Then the slides were transferred to wash solution 2 (0.06× SSPE, 0.5% N-Lauroylsarcosine) and incubated 1 minute. After this, the slides were transferred to an acetonitrile bath (Sigma, order number A3396) and incubated for 1 minute. At last the rack was transferred to Agilent stabilization and drying solution (Agilent, order number 5185–5979) and incubated for 30 seconds.

### Scan and Data analysis of Agilent microarrays

Microarray slides were scanned with an Agilent microarray scanner and the scans were quantified with the Agilent Feature Extraction software 8.5.1.1. Background subtraction and dye normalization for each array was performed within the Agilent Feature Extraction software with default settings. The quantified data was subsequently loaded into GeneSpring 7.3.1 for further analysis. Expression data from the dye-swap pairs were averaged in order to eliminate any potential gene-specific dye effect. Probes with signals close to background (average signal<500) as well as probes which were flagged as saturated by the Agilent Feature Extraction Software were excluded. From the remaining probes the average ratio of MCF positive vs MCV negative animals was computed.

### Real-time PCR

The Taqman real-time PCR for detection of OvHV-2 DNA in animal samples was used essentially as described previously [23]. Primers and probe are listed in Table 5.

**Table 5:** Oligonucleotides used.

Oligonucleotide	Sequence (5′ to 3′)
OvHV-2 forward	TGG TAG GAG CAG GCT ACC GT
OvHV-2 reverse	ATC ATG CTG ACC CCT TGC AG
OvHV-2 probe	[6FAM]-TCC ACG CCG TCC GCA CTG TAA GA
LANA2-F	GTG GAG CGT TAG GAT TGA GC
LANA2-R	CAG GGC AAA ACG TAA AAA GC
Ov9-F	CGG GAC CAT TAC AAG AAG
Ov9-R	GCA TAA CAG AAG CAT AGC
Intergenic-F	GTG TGG TGA CAC ATT CCC AG
Intergenic-R	ATG TAA GAC CCC TTA GCC CC
ORF25-F	ACT GCG GAC GTG GCC TAC TT
ORF25-R	GTC CAG GAG GGC TCG GTG TG

### qRT-PCR

Quantitative two-step RT-PCRs were established (specificity, sensitivity, efficiency) for ORF73, Ov9, and intergenic RNA as described previously [82]. Briefly, RNA was extracted and purified from contaminating DNA as described above. The QScript cDNA Supermix (Quanta Biosciences, VWR International, Dietikon, Switzerland) was used for cDNA synthesis (30 min at 42°C before inactivating the RT at 85°C for 5 min) in a volume of 20 µl. For control reactions the same amounts of RNA template were diluted to the same volume in RNase/DNase-free water and kept on ice during the RT cycle. In the second step, 1 µl of cDNA (or RNA) was mixed with 10 µl Perfecta SYBR Fastmix (Quanta) and appropriate amounts of primers (100 nM final concentration; Table 5) before being brought to a volume of 20 µl. The following cycles were run to yield a detection limit of between 10 and 100 copies per sample: 10 min at 95°, 40 cycles of 5 sec at 95° and 20 sec at 60° (only 58.3° for ORF73). The melt curve was run immediately after amplification, starting at 50°C and increasing the temperature for 80 cycles by 0.5°C every 10 seconds. Sensitivity and efficiency were established by using decreasing concentrations of cloned DNA templates, whereas controls for specificity included templates from unrelated viruses [82], [83].

### Conventional RT-PCR

Conventional RT-PCR for ORF25 was performed as described by others [40]. The templates and controls used here were the same as for qRT-PCR described above.

### cRNA preparation for the Affymetrix microarray

Total RNA samples (2 µg) were reverse-transcribed into double-stranded cDNA, in vitro transcribed in presence of biotin-labeled nucleotides using a IVT Labeling Kit (Affymetrix Inc., P/N 900449, Santa Clara, CA), purified and quantified using BioRobot Gene Exp–cRNA Target Prep (Qiagen AG, Switzerland). The labeled cRNA quality was determined using a Bioanalyzer 2100.

### Hybridization of the Affymetrix microarray

Biotin-labeled cRNA samples (10 µg) were fragmented randomly to 35–200 bp at 94°C in Fragmentation Buffer (Affymetrix inc., P/N 900371) and were mixed in 300 µl of Hybridization Mix (Affymetrix Inc., P/N 900720) containing Hybridization Controls and Control Oligonucleotide B2 (Affymetrix Inc., P/N 900454), before hybridization to GeneCHIP® Bovine Genome arrays (Affymetrix Inc., P/N 900561) for 16 hours at 45°C was performed. Arrays were then washed using an Affymetrix Fluidics Station 450 (FS450_002 protocol. An Affymetrix GeneChip Scanner 3000 (Affymetrix Inc.) was used to measure the fluorescent intensity emitted by the labeled target.

### Statistical analysis of the Affymetrix microarray

Raw data processing was performed using the Affymetrix GCOS 1.4 software (Affymetrix Inc.). After hybridization and scanning, probe cell intensities were calculated and summarized for the respective probe sets by means of the MAS5 algorithm [84]. To compare the expression values of the genes from chip to chip, global scaling was performed, which resulted in the normalization of the trimmed mean of each chip to a target intensity (TGT value) of 500 as detailed in the statistical algorithms description document of Affymetrix (Affymetrix, 2002, see also [85]). Quality control measures were considered before performing the statistical analysis. These included adequate scaling factors (between 1 and 3 for all samples) and appropriate numbers of present calls calculated by application of signed-rank call algorithm [86]. The efficiency of the labeling reaction and the hybridization performance was controlled with the following parameters: present calls and optimal 3′/5′ hybridization ratios (around 1) for the housekeeping genes (GAPDH and ACO7), for the poly A spike in controls and the prokaryotic control (BIOB, BIOC, CREX, BIODN).

Differential transcript abundance was identified as follows: Probes that were absent in the uninfected and the infected animals were excluded. We considered a probe absent if it had more than one absent call among the infected animals and more than one absent call among the uninfected animals. Student's t-test was applied to test the present probes for significant infection-induced higher or lower abundance. The magnitude of the change was computed from the averaged values of the infected and uninfected animals.

### Isolation of lymphocytes

For the isolation of lymphatic cells from lymphnodes, fat and connective tissue were removed and the remaining tissue was cut into small pieces and filtered through a sieve with a mesh size of 1 mm before being suspended in phosphate buffered saline solution (PBS). After washing tree times with 50 ml PBS, low speed centrifugation, and re-filtering for removal of aggregates, the cells were resuspended in 10 ml PBS and filtered trough a cell strainer with a mesh size of 100 µm (BD Falcon, Bedford, MA, USA).

### Fluorescence-activated flow cytometry

CD4 and CD8 subsets were determined in combination with CD2 staining. Anti bovine CD4 (CACT138A), anti bovine CD8 (CACT80C) and anti bovine CD2 (16-1E10) were from VMRD, Inc, Pullmann, WA, USA). For staining, 100 µl EDTA blood or 100 µl isolated lymphnode cells (10^6^) were added to 5 µl of pre-diluted antibody (1/100). After incubation for 30 min at 4°C, 2 ml of erythrocyte lysing solution (8.29 g/l NH_4_Cl, 1 g/l KHCO_3_, 37 mg/l Na_2_ EDTA) were added. After 3 minutes at ambient temperature, cells were pelleted by centrifugation (350×g). The supernate was discarded and the cells resuspended in 200 µl of PBS supplemented with 1% fetal calf serum (FCS) and the secondary antibodies (APC labeled anti mouse IgG1 from BD Pharmingen, BD Biosciences, San Jose, CA, USA and goat anti mouse IgG2a-FITC from Southern Biotech, Birmingham, AL, USA) diluted 1/1000. After 30 min at 4°C, cells were washed with 2 ml PBS and resuspended in 250 µl PBS with 1% FCS. Finally, cells were analyzed in a FACScalibur (BD Biosciences, San Jose, CA, USA). A gate was set to the region corresponding to the lymphocytes, based on the forward and side scatter diagram. A minimum of 1000 gated events were acquired and analyzed. FL1 and FL4 double positive cells were counted.
